# LASSO regression-derived first-trimester (9–14^+6^ weeks) risk stratification model for gestational diabetes mellitus: development, validation, and open-access web tool in a retrospective Chinese cohort

**DOI:** 10.1007/s00404-026-08353-y

**Published:** 2026-03-11

**Authors:** Xiao Wang, Jin Zhang, Shitong Zhan, Xianming Xu

**Affiliations:** Department of Obstetrics and Gynaecology, Shanghai General Hospital, Shanghai Jiao Tong University School of Medicine, Shanghai, 201,600 China

**Keywords:** Gestational diabetes mellitus, Early prediction, LASSO regression, Nomogram, Web-based calculator, Metabolic biomarkers, Risk stratification

## Abstract

**Background:**

Early identification of gestational diabetes mellitus (GDM) is critical for mitigating adverse maternal and neonatal outcomes. Existing prediction models face limitations in clinical utility due to inconsistent variable selection and reliance on impractical biomarkers. This study aimed to develop and validate a resource-efficient GDM prediction model using routinely available first-trimester clinical indicators and deploy it as an open-access web tool.

**Methods:**

A retrospective cohort of 1818 pregnancies from a Shanghai tertiary hospital (2023) was randomly divided into training (70%) and validation (30%) sets. Three predictor screening strategies (traditional logistic regression, least absolute shrinkage and selection operator (LASSO) regression with 1SE rule, and LASSO–MIN rule) were compared. The model performance was assessed by the area under the receiver operating characteristic (ROC) curves (AUC), the calibration curve, the clinical decision curve (DCA) and the clinical impact curve (CIC). The optimal model was visualized as a nomogram and deployed as an open access web calculator.

**Results:**

The LASSO–1SE model achieved the best balance of accuracy and simplicity, with an AUC of 0.717 (95% CI 0.681–0.753), sensitivity 69.7%, specificity 64.9%, and high positive predictive value (PPV = 92.3%).The model showed robust calibration (Hosmer–Lemeshow *P* > 0.3) and clinical utility across risk thresholds in DCA and CIC. A nomogram and an open-access web calculator (https://wangxiao0922.shinyapps.io/20250309/) were developed for risk stratification.

**Conclusions:**

This resource-efficient tool enables early GDM risk stratification using routine clinical variables, supporting timely intervention in diverse healthcare settings.

## What does this study adds to the clinical work

**Table:** 

This study developed a web-based, resource-efficient tool that uses routinely available dinical indicators from the first trimester to stratify the risk of gestational diabetes mellitus. It enables early identification of high-risk pregnancies, facilitating timely intervention at least 10 weeks earlier than the standard OGTT diagnosis.	

## Background

Gestational diabetes mellitus (GDM) is characterized by hyperglycemia first detected during pregnancy at glucose concentrations below the diagnostic threshold for overt diabetes. GDM affects 14–27.6% of pregnancies globally, with rising incidence linked to obesity and type 2 diabetes(T2DM) [1, 2],with variations in prevalence influenced by risk factors, screening protocols, and diagnostic criteria [1].Globally, the international association of diabetes and pregnancy study groups (IADPSG) estimates a GDM prevalence of 14.0% [3], while mainland China reports a rate of 14.8% [4].This condition poses significant maternal and neonatal risks, including pre-eclampsia, cesarean delivery, fetal macrosomia, neonatal hypoglycemia, and long-term cardiometabolic sequelae for both mother and offspring [5–9].Alarmingly, up to 31% of T2DM cases in parous women are attributable to prior GDM [10],underscoring the critical need for early post-pregnancy interventions to mitigate diabetes risk [11].

Early lifestyle modifications have demonstrated efficacy in reducing T2DM incidence and improving metabolic outcomes in offspring [12]. Currently, the diagnosis of GDM relies on a ‘one-step’ 75 g OGTT at 24–28 weeks of gestation [3],which may delay intervention, as insulin resistance and *β*-cell dysfunction often manifest earlier in pregnancy [13].This diagnostic lag highlights the potential of early risk stratification to guide timely interventions, such as intensive diet-exercise regimens and weight management, which effectively reduce GDM incidence in high-risk cohorts [14–17].Consequently, integrating multidimensional biomarkers—including metabolic, genetic, and hematological parameters—into early screening strategies is imperative for optimizing maternal and infant health [14, 18].

Existing prediction models for gestational diabetes mellitus (GDM) that integrate early pregnancy metabolic markers (e.g., HbA1c and lipid profiles) and clinical history face limitations in clinical utility due to inconsistencies in risk factor selection, variability in study quality, and reliance on complex or impractical biomarkers [19–21]. While machine learning (ML) approaches, particularly multi-omics integration (e.g., metabolomics and gut microbiome), demonstrate potential for improving predictive accuracy [19–21], their superiority over traditional logistic regression remains contentious [22, 23].This underscores the necessity for model selection strategies that prioritize clinical feasibility and interpretability.

To address these challenges, this study aims to develop an early GDM risk prediction tool utilizing routinely collected obstetric history and clinical indicators from the first trimester. We systematically compare traditional statistical methods with LASSO regression—a regularization technique derived from machine learning—to identify an optimal modeling approach. The clinical utility of the final model is rigorously validated through decision curve analysis (DCA) and clinical impact curve (CIC). Furthermore, key predictors are translated into a quantifiable risk score and implemented as an open-access web-based calculator. This resource-efficient solution seeks to enhance early GDM screening and enable targeted prenatal interventions, thereby improving maternal and neonatal health outcomes.

## Methods

### Participant’s information

This retrospective cohort study encompassed pregnant women who delivered at the southern part of Shanghai General Hospital, affiliated with Shanghai Jiao Tong University School of Medicine, from January 1 to December 31, 2023. The inclusion criteria were as follows: (1) completion of regular prenatal care and initial laboratory testing at the study center; (2) singleton live birth; and (3) absence of pre-existing chronic cardiovascular, hepatic, or renal diseases. The exclusion criteria included multiple pregnancies, foreign nationality, maternal age less than 18 years or greater than 45 years, pre-pregnancy diabetes mellitus or diagnosis of diabetes mellitus during the first or second trimester of pregnancy, neonatal death, absence of an oral glucose tolerance test (OGTT) at 24–28 weeks of gestation, initial carding not performed at 9 to 14^+6^ weeks of gestation, or missing clinically/laboratory significant data. After applying the inclusion and exclusion criteria, a total of 1818 participants (259 with GDM and 1559 with normal glucose tolerance [NGT]) were included, resulting in a GDM incidence of 14.25%. Utilizing a random seed (123) in R version 4.4.2, the cohort was divided into training (70%, *n* = 1274; 192 GDM cases) and validation (30%, *n* = 544; 67 GDM cases) sets. Ethical approval for the study was obtained from the Ethics Committee of Shanghai General Hospital (approval number: 2025KS083). Being a retrospective study, informed consent was waived by the ethics committee and adhered to the declaration of Helsinki. The details of our study process are depicted in the Flowchart.
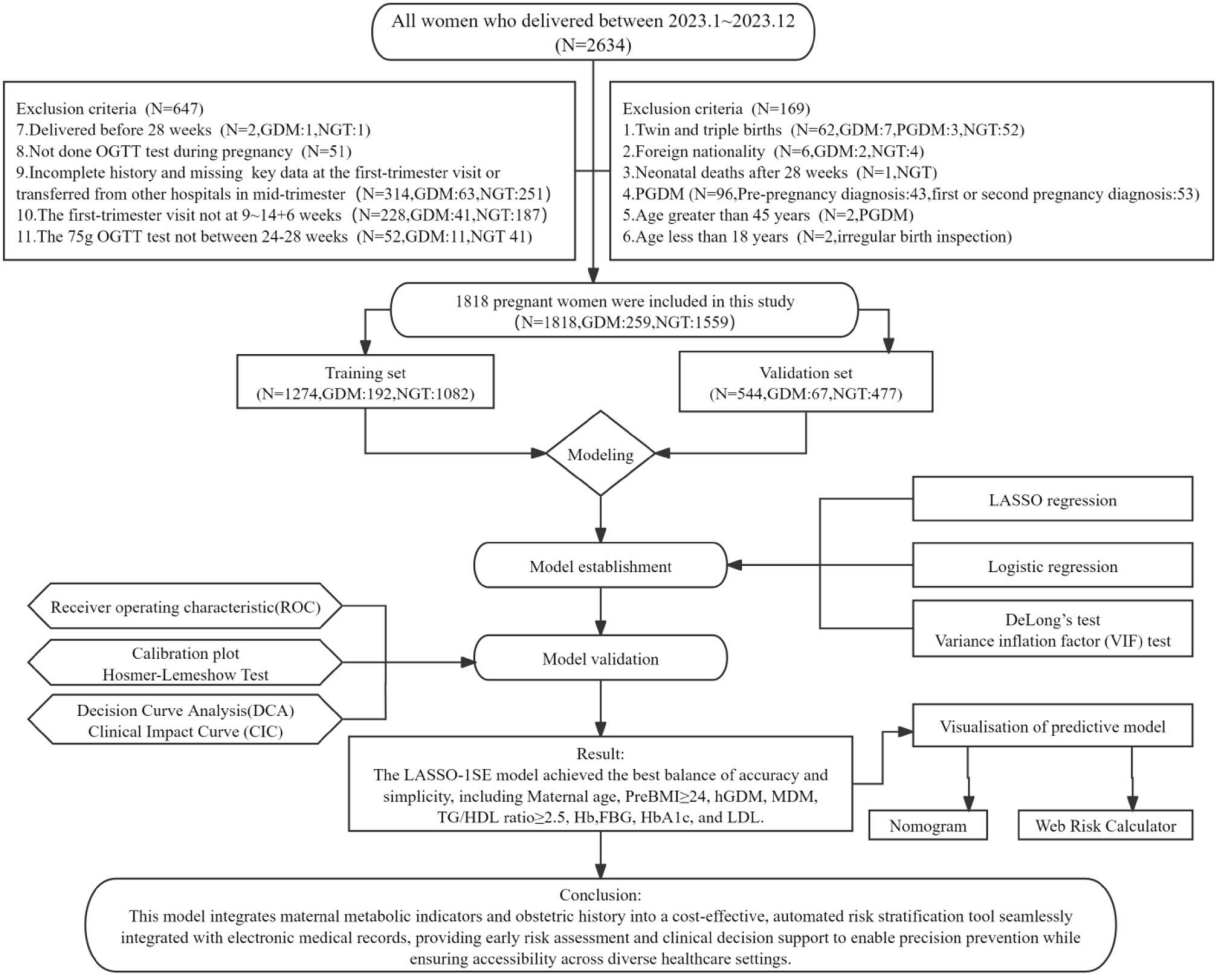


### Definitions and diagnostic criteria

GDM was diagnosed at 24–28 weeks using the IADPSG criteria via a one-step 75 g OGTT [3]. Diagnosis required meeting ≥ 1 of the following thresholds: fasting glucose ≥ 5.1 mmol/L, 1-h glucose ≥ 10.0 mmol/L, or 2-h glucose ≥ 8.5 mmol/L. Pregnancy with diabetes mellitus (PGDM) was excluded based on pre-pregnancy diabetes history or meeting any of the following criteria during the first or second trimester of pregnancy: fasting glucose ≥ 7.0 mmol/L, 2-h glucose ≥ 11.1 mmol/L, random glucose ≥ 11.1 mmol/L with hyperglycemic symptoms, or glycated hemoglobin (HbA1c) ≥ 6.5% [24].

### Data collection

All pregnant women were given a detailed history by a specialized nurse at their initial visit, and an electronic history was taken. At the time of history collection, the following characteristics were obtained by reviewing the electronic medical records from the early pregnancy clinic and the history written by the obstetrician:

- **Demographic information:** Maternal age, geographic region, and educational level.
- **Clinical history:** Gravidity, parity, history of GDM (hGDM), history of previous macrosomia, conception method, polycystic ovary syndrome (PCOS), chronic hypertension, thyroid diseases, history of diabetes in first-degree relatives, etc.
- **Clinical parameters:** Systolic blood pressure (SBP), diastolic blood pressure (DBP), and PreBMI calculated as self-reported pre-pregnancy weight (kg)/height (m)^2^.
- **First-trimester data(9 ~ 14**^**+6**^** weeks):** Hb1, ferritin(SF1),FBG1,HbA1c1,total cholesterol (TC1), triglycerides (TG1), high-density lipoprotein cholesterol (HDL1), and LDL1.
- **Second-trimester data(24 ~ 28 weeks):** Hb2, SF2, FBG2, 1-h blood glucose (1 h-BG), 2-h blood glucose (2 h-BG), fasting insulin (FIN), 1-h insulin (1 h-FIN), 2-h insulin (2 h-FIN), HbA1c2,glycosylated albumin (GA),TC2,TG2,HDL2 and LDL2 and information on maternal and paediatric birth outcomes.

Since fasting insulin was not tested in early pregnancy in most pregnant women, the homeostasis model assessment of insulin resistance (HOMA–IR) was only presented at 24–28 weeks. Insulin function was assessed using the TyG index and TG/HDL-C ratio as metabolic markers [25].HOMA–IR was calculated as (FBG [mmol/L]) × (FIN [µU/mL])/22.5 to represent the insulin resistance [26].The triglyceride–glucose (TyG) index was calculated as ln (fasting TG [mg/dL] × FBG [mg/dL]/2).

### Statistical methods

Data were analyzed using SPSS 27.0 (IBM) and R 4.4.2(R foundation for statistical computing).To build a robust and clinically interpretable prediction model, we adopted a machine learning-integrated analytical strategy. First, LASSO regression was employed using the glmnet package to perform automated variable selection from the initial candidate predictors. Compared with traditional stepwise regression, LASSO incorporates regularization, which reduces overfitting, improves model stability, and strengthens generalizability—key advantages of machine learning for handling multidimensional clinical data. Although the final model retains a linear form for clinical interpretability, this framework inherently supports the exploration of complex, nonlinear relationships, a capacity beyond conventional regression analysis.

The variables selected by LASSO were then incorporated into a multivariable logistic regression model (stats package) to quantify the risk of GDM. Multicollinearity was assessed using the variance inflation factor (VIF; car package). To translate the model into a clinically usable tool, it was presented visually as a nomogram and implemented as an open-access web-based calculator, facilitating straightforward and early risk assessment at the point of care.

Normality was assessed via Shapiro–Wilk tests and the shape of the histograms. Continuous variables were reported as mean ± SD (normal or approximately normal distribution) or median (Q1, Q3) (non-normal), compared using Student’s *t* test or Mann–Whitney *U* test. Categorical variables were expressed as frequencies and percentages (%), and were compared via *χ*^2^ or Fisher’s exact tests. There were no missing values in the disaggregated data, with less than 1% of continuous variables missing in the first trimester and less than 5% missing in the second trimester. Missing values were imputed using the mean (for normally distributed variables) or median (for non-normal variables) from five imputations. A two-tailed *p* value < 0.05 was considered statistically significant.

### Sample size estimation

The minimum sample size was calculated using the events per predictor (EPP = 20) criterion [27, 28], with the following parameters: GDM prevalence≈14.5%, 10 predictors, target AUC = 0.7–0.8. The formula applied was:

$$N = \frac{{\left( {EPP \times Number of predictors} \right)}}{GDM prevalence} = \frac{{\left( {20 \times 10} \right)}}{0.145} \approx 1380$$

Our sample size (1818) met this requirement.

### Model construction

Three models were constructed using three variable screening methods:

- **Model_1**: Variables were screened by univariate analysis (*p* < 0.05), VIF < 5 and backward stepwise regression, and finally MDM (Mother has diabetes mellitus), PreBMI ≥ 24, TG/HDL1 ratio ≥ 2.5, Hb1, HbA1c1, FBG1, hGDM were included.
- **Model_2**: Screening variables based on the‘1 standard error (1SE)’ rule (*λ* = 0.0239) of LASSO regression, and finally included Model_1 variables, LDL1 and age.
- **Model_3**: Initial screening variables based on the ‘minimum error (MIN)’ rule (*λ* = 0.0065) of LASSO regression, then optimized by multivariate logistic regression, and finally incorporating Model_2 variables + Parity.

**Fig. 1:**
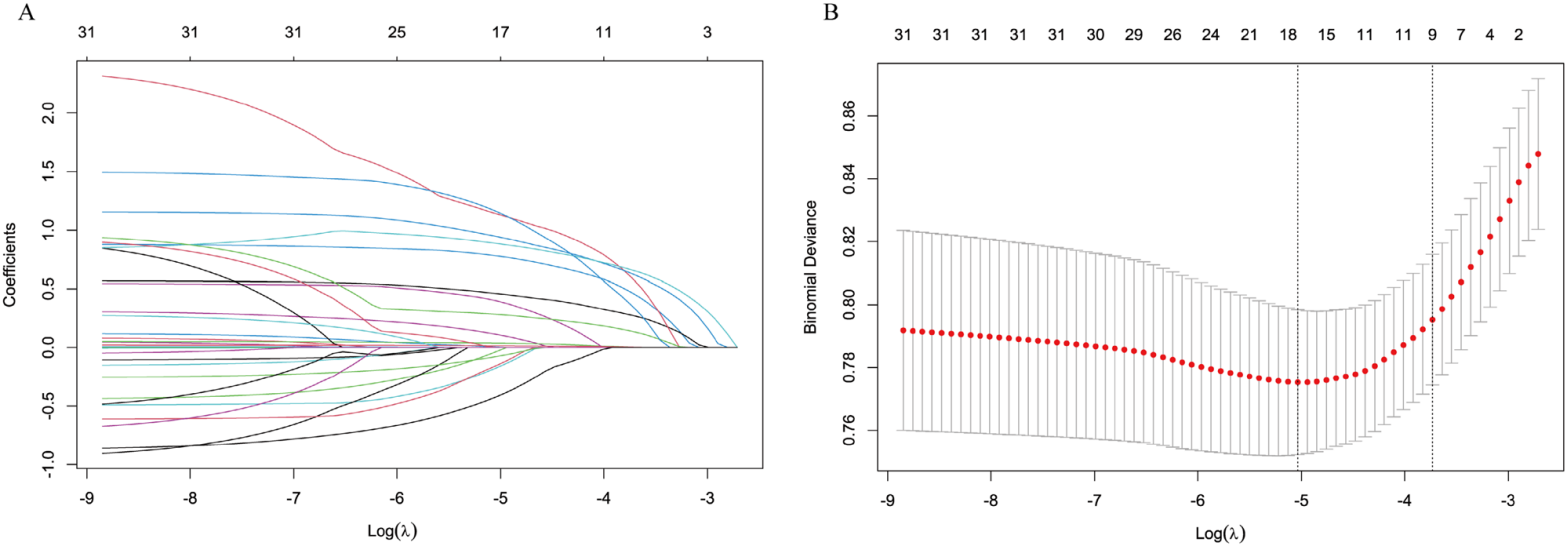
**A**: LAACO coefficient paths showing the variables with non-zero coefficients selected at the optimum λ. **B**: tenfold cross-validation curves: vertical dashed lines indicate log (*λ*) values for ‘1SE’ (*λ* = 0.0239, right) and ‘MIN’ (*λ* = 0.0065, left)

The trajectories of the LASSO coefficients for different values of *λ* are visualized in Fig. 1. Model performance was assessed using AUC, calibration curves, DeLong’*s* test (in Table 4), and Hosmer–Lemeshow tests. Based on discrimination and calibration metrics, Model_2 was chosen as the final model, with subsequent development of a nomogram and web tool for clinical risk visualization. LASSO-selected variables’ contributions were visualized via bar plots to improve interpretability. DCA and CIC further evaluated net benefit and clinical utility.

## Results

### Participant characteristics

The study included 1818 participants, with a GDM prevalence of 14.25%, consistent with prior reports [1, 4]. The cohort was divided into training (*n* = 1274) and validation (*n* = 544) sets, with balanced demographics, obstetric history, and clinical/laboratory parameters between the two groups (Table 1, *p* > 0.05 for all comparisons).

**Table 1:** Baseline characteristics of participants in training and validation cohorts

Characteristics	Total (*n* = 1818)	Train (*n* = 1274)	Validation (*n* = 544)	*t*/*χ*^2^/*Z*	*P* value
	Maternal baseline information				
Age (years)	30.84 ± 4.00	30.87 ± 3.98	30.77 ± 4.04	*t* = 0.48	0.632
PreBMI ≥ 24 kg/m^2^	377 (20.74)	274 (21.51)	103 (18.93)	*χ*^2^ = 1.54	0.215
Region				*χ*^2^ = 0.01	0.917
South	1384 (76.13)	969 (76.06)	415 (76.29)		
North	434 (23.87)	305 (23.94)	129 (23.71)		
Education				*χ*^2^ = 0.23	0.634
High school and below	385 (21.18)	266 (20.88)	119 (21.88)		
College and above	1433 (78.82)	1008 (79.12)	425 (78.12)		
Minority(Yes)	48 (2.64)	30 (2.35)	18 (3.31)	*χ*^2^ = 1.35	0.245
FHBP(Yes)	325 (17.88)	223 (17.50)	102 (18.75)	*χ*^2^ = 0.40	0.525
MHBP(Yes)	274 (15.07)	186 (14.60)	88 (16.18)	*χ*^2^ = 0.74	0.390
FDM(Yes)	93 (5.12)	68 (5.34)	25 (4.60)	*χ*^2^ = 0.43	0.511
MDM(Yes)	81 (4.46)	54 (4.24)	27 (4.96)	*χ*^2^ = 0.47	0.493
SBP (mmHg)	111.51 ± 11.15	111.23 ± 11.22	112.17 ± 10.99	*t* = − 1.65	0.100
DBP (mmHg)	69.44 ± 8.32	69.31 ± 8.45	69.72 ± 8.02	*t* = − 0.96	0.337
VT1 (kg/week)	0.12 ± 0.20	0.11 ± 0.20	0.13 ± 0.20	*t* = − 1.67	0.096
IVF(Yes)	131 (7.21)	92 (7.22)	39 (7.17)	*χ*^2^ = 0.00	0.969
PCOS (Yes)	57 (3.14)	37 (2.90)	20 (3.68)	*χ*^2^ = 0.75	0.387
Thyroid diseases(Yes)	409 (22.50)	286 (22.45)	123 (22.61)	*χ*^2^ = 0.01	0.940
Autoimmune(Yes)	12 (0.66)	6 (0.47)	6 (1.10)	*χ*^2^ = 1.46	0.227
Hypertension(Yes)	22 (1.21)	16 (1.26)	6 (1.10)	*χ*^2^ = 0.07	0.785
Gravida				*χ*^2^ = 0.58	0.901
1	761 (41.86)	536 (42.07)	225 (41.36)		
2	544 (29.92)	378 (29.67)	166 (30.51)		
3	313 (17.22)	223 (17.50)	90 (16.54)		
≥ 4	200 (11.00)	137 (10.75)	63 (11.58)		
Parity				*χ*^2^ = 1.64	0.441
0	1133 (62.32)	795 (62.40)	338 (62.13)		
1	611 (33.61)	432 (33.91)	179 (32.90)		
≥ 2	74 (4.07)	47 (3.69)	27 (4.96)		
History of GDM (hGDM)				*χ*^2^ = 1.28	0.274
Nullipara and Multipara without hGDM	1789 (98.40)	1251 (98.19)	538 (98.90)		
Multipara with hGDM	29 (1.60)	23 (1.81)	6 (1.10)		
History of macrosomia				*χ*^2^ = 0.23	0.662
Nullipara and Multipara without macrosomia	1777 (97.74)	1244 (97.65)	533 (97.98)		
Multipara with macrosomia	41 (2.26)	30 (2.35)	11 (2.02)		
History of APO(Yes)	345 (18.98)	249 (19.54)	96 (17.65)	*χ*^2^ = 0.89	0.345
	*Laboratory data between 9* ~ *14*^+*6*^* weeks of pregnancy*				
TyG1 index	8.40 ± 0.38	8.41 ± 0.39	8.38 ± 0.37	*t* = 1.55	0.122
TG/HDL1 ratio ≥ 2.5(Yes)	24 (1.32)	19 (1.49)	5 (0.92)	*χ*^2^ = 0.96	0.328
Hb1 (g/L)	127.00 ± 9.90	126.79 ± 10.10	127.49 ± 9.39	*t* = − 1.37	0.170
SF1 (ng/ml)	44.30 (24.95,74.57)	44.40 (25.30,75.72)	43.50 (24.80,71.80)	*Z* = − 0.61	0.543
HbA1c1 (%)	5.22 ± 0.28	5.22 ± 0.29	5.22 ± 0.26	*t* = 0.05	0.963
FBG1 (mmol/L)	4.36 ± 0.40	4.37 ± 0.41	4.34 ± 0.38	*t* = 1.40	0.163
TC1 (mmol/L)	4.62 ± 0.77	4.63 ± 0.77	4.61 ± 0.77	*t* = 0.37	0.708
TG1 (mmol/L)	1.28 (1.03,1.61)	1.28 (1.03,1.61)	1.26 (1.03,1.60)	*Z* = − 0.81	0.416
HDL1 (mmol/L)	1.61 ± 0.34	1.61 ± 0.34	1.62 ± 0.34	*t* = − 0.74	0.458
LDL1 (mmol/L)	2.48 ± 0.67	2.49 ± 0.68	2.45 ± 0.65	*t* = 0.91	0.365
	*Prevalence of GDM*				
GDM(Yes)	259 (14.25)	192 (15.07)	67 (12.32)	*χ*^2^ = 2.37	0.124

*HBP* Hypertension, *FHBP* Father has HBP, *MHBP* Mother has HBP, DM Diabetes mellitus, *FDM* Father has DM, *MDM* Mother has DM, *IVF* In Vitro fertilization, *APO* Adverse pregnancy outcomes, *VT1* Weight velocity in first trimester = (Weight1–Weight0)/week of gestation at first obstetric examination

Comparative analyses between GDM and NGT groups revealed consistent risk factors across both cohorts (Table 2). Women with GDM exhibited significantly higher maternal age (training: 31.79 vs. 30.71 years; validation: 32.12 vs. 30.58 years, *p* < 0.05), elevated PreBMI (≥ 24 kg/m^2^: training, 35.42% vs. 19.04%; validation, 35.82% vs. 16.56%, *p* < 0.05), and impaired metabolic profiles, including higher TyG index, TG/HDL ratio, HbA1c, and FBG (all *p* < 0.05). A history of GDM strongly predicted recurrence (training: *p* < 0.001; validation: *p* = 0.027), and SBP was elevated in GDM cases (*p* < 0.05). No significant differences were observed in region, education level, thyroid function, or chronic hypertension. These findings underscore the critical roles of dysregulated glucose–lipid metabolism, advanced maternal age, obesity, and prior GDM history in identifying high-risk populations.

**Table 2:** Comparative analysis between women with GDM and those with NGT across training and validation cohorts

Characteristics	Train			Validation		
	GDM	NGT	*P*	GDM	NGT	*P*
*N* (%)	192 (15.07)	1082 (84.93)		67 (12.32)	477 (87.68)	
*Maternal baseline information*						
Age (years)	31.79 ± 4.06	30.71 ± 3.94	< .001	32.12 ± 4.00	30.58 ± 4.02	0.004
PreBMI ≥ 24 kg/m^2^	68 (35.42)	206 (19.04)	< .001	24 (35.82)	79 (16.56)	< .001
Region			0.995			0.117
South	146 (76.04)	823 (76.06)		46 (68.66)	369 (77.36)	
North	46 (23.96)	259 (23.94)		21 (31.34)	108 (22.64)	
Education			0.834			0.291
High school and below	39 (20.31)	227 (20.98)		18 (26.87)	101 (21.17)	
College and above	153 (79.69)	855 (79.02)		49 (73.13)	376 (78.83)	
Minority(Yes)	3 (1.56)	27 (2.50)	0.598	3 (4.48)	15 (3.14)	0.836
FHBP (Yes)	33 (17.19)	190 (17.56)	0.900	18 (26.87)	84 (17.61)	0.069
MHBP(Yes)	35 (18.23)	151 (13.96)	0.122	11 (16.42)	77 (16.14)	0.954
FDM (Yes)	11 (5.73)	57 (5.27)	0.793	2 (2.99)	23 (4.82)	0.718
MDM (Yes)	20 (10.42)	34 (3.14)	< .001	5 (7.46)	22 (4.61)	0.480
SBP (mmHg)	112.77 ± 11.18	110.95 ± 11.21	0.039	115.61 ± 12.08	111.68 ± 10.75	0.006
DBP (mmHg)	70.49 ± 8.51	69.10 ± 8.42	0.036	71.39 ± 9.12	69.49 ± 7.84	0.070
VT1 (kg/week)	0.14 ± 0.20	0.11 ± 0.20	0.079	0.17 ± 0.22	0.12 ± 0.20	0.060
IVF (Yes)	21 (10.94)	71 (6.56)	0.031	3 (4.48)	36 (7.55)	0.510
PCOS (Yes)	6 (3.12)	31 (2.87)	0.843	2 (2.99)	18 (3.77)	1
Thyroid diseases (Yes)	44 (22.92)	242 (22.37)	0.866	14 (20.90)	109 (22.85)	0.720
Autoimmune (Yes)	1 (0.52)	5 (0.46)	1	1 (1.49)	5 (1.05)	0.547
Hypertension (Yes)	2 (1.04)	14 (1.29)	1	1 (1.49)	5 (1.05)	0.547
Gravida			0.682			0.226
1	76 (39.58)	460 (42.51)		24 (35.82)	201 (42.14)	
2	56 (29.17)	322 (29.76)		17 (25.37)	149 (31.24)	
3	35 (18.23)	188 (17.38)		15 (22.39)	75 (15.72)	
≥ 4	25 (13.02)	112 (10.35)		11 (16.42)	52 (10.90)	
Parity			0.217			0.024
0	125 (65.10)	670 (61.92)		32 (47.76)	306 (64.15)	
1	64 (33.33)	368 (34.01)		29 (43.28)	150 (31.45)	
≥ 2	3 (1.56)	44 (4.07)		6 (8.96)	21 (4.40)	
History of GDM (hGDM)			< .001			0.027
Nullipara and Multipara without hGDM	182 (94.79)	1069 (98.80)		64 (95.52)	474 (99.37)	
Multipara with hGDM	10 (5.21)	13 (1.20)		3 (4.48)	3 (0.63)	
History of macrosomia			0.613			0.893
Nullipara and Multipara without macrosomia	186 (96.88)	1058 (97.78)		65 (97.01)	468 (98.11)	
Multipara with macrosomia	6 (3.12)	24 (2.22)		2 (2.99)	9 (1.89)	
History of APO(Yes)	45 (23.44)	204 (18.85)	0.140	11 (16.42)	85 (17.82)	0.778
*Laboratory data between 9* ~ *14*^+*6*^* weeks of pregnancy*						
TyG1 index	8.53 ± 0.39	8.39 ± 0.38	< .001	8.49 ± 0.33	8.37 ± 0.37	0.014
TG/HDL1 ratio ≥ 2.5(Yes)	9 (4.69)	10 (0.92)	< .001	1 (1.49)	4 (0.84)	0.483
Hb1 (g/L)	129.00 ± 10.33	126.40 ± 10.02	0.001	129.18 ± 9.97	127.25 ± 9.29	0.116
SF1 (ng/ml)	48.25 (28.08, 77.17)	43.90 (24.90, 75.47)	0.261	42.40 (22.25, 59.95)	44.08 (25.70, 72.00)	0.376
HbA1c1 (%)	5.33 ± 0.31	5.20 ± 0.28	< .001	5.31 ± 0.28	5.21 ± 0.25	0.002
TC1 (mmol/L)	4.79 ± 0.80	4.60 ± 0.76	0.002	4.53 ± 0.65	4.63 ± 0.78	0.321
TG1 (mmol/L)	1.34 (1.10, 1.67)	1.26 (1.02, 1.59)	0.007	1.37 (1.12, 1.58)	1.24 (1.00, 1.60)	0.092
HDL1 (mmol/L)	1.59 ± 0.34	1.61 ± 0.34	0.359	1.60 ± 0.34	1.63 ± 0.34	0.582
LDL1 (mmol/L)	2.68 ± 0.77	2.45 ± 0.66	< .001	2.41 ± 0.55	2.46 ± 0.66	0.553
FBG1 (mmol/L)	4.55 ± 0.46	4.34 ± 0.39	< .001	4.58 ± 0.42	4.31 ± 0.36	< .001

### OGTT results in the second trimester and comparison of pregnancy outcomes

Table 2 demonstrates the baseline characteristics of women with gestational diabetes mellitus (GDM) and normal glucose tolerance (NGT) in the training and validation cohorts. No significant differences were observed in demographic features, metabolic parameters, or obstetric history between the two groups (all *p* > 0.05), confirming the balanced distribution of the data sets. Building on this, Table 3. further evaluates the metabolic profiles and maternal–neonatal outcomes between GDM and NGT in the total cohort during the second trimester (24–28 weeks of gestation). Women with GDM exhibited pronounced metabolic dysregulation, characterized by elevated TyG index (9.04 vs. 8.88, *p* < 0.001)—a marker linked to increased GDM risk in early pregnancy [29]—along with higher HbA1c (5.10% vs. 4.94%, *p* < 0.001), elevated fasting and postprandial glucose levels (e.g., 2 h-BG: 8.14 vs. 6.15 mmol/L, *p* < 0.001), and greater insulin resistance (HOMA–IR: 2.32 vs. 1.63, *p* < 0.001).

**Table 3:** OGTT results in the second trimester and comparison of pregnancy outcomes

Variables	Total (*n* = 1818)	GDM (*n* = 259)	NGT (*n* = 1559)	*t*/*χ*^2^/*Z*	*P*
*Laboratory data between 24* ~ *28 weeks of pregnancy*					
TyG2 index	8.90 ± 0.37	9.04 ± 0.38	8.88 ± 0.37	*t* = -6.53	< 0.001
TG/HDL2 ratio ≥ 2 5 (Yes)	79 (4.35)	20 (7.72)	59 (3.78)	*χ*^2^ = 8.28	0.004
Hb2 (g/L)	114.27 ± 9.12	116.18 ± 8.87	113.95 ± 9.12	*t* = -3.65	< 0.001
SF2 (ng/mL)	11.90 (7.50–20.50)	12.60 (7.70–22.20)	11.80 (7.50–20.30)	*Z* = − 1.40	0.161
HbA1c2 (%)	4.96 ± 0.31	5.10 ± 0.33	4.94 ± 0.29	*t* = − 7.97	< 0.001
GA (%)	12.05 ± 1.05	12.28 ± 1.06	12.01 ± 1.04	*t* = − 3.84	< 0.001
TC2 (mmol/L)	5.93 ± 0.99	5.85 ± 1.04	5.94 ± 0.98	*t* = 1.44	0.149
TG2 (mmol/L)	2.11 (1.70–2.62)	2.17 (1.79–2.68)	2.09 (1.69–2.62)	*Z* = − 2.24	0.025
HDL2 (mmol/L)	1.91 ± 0.41	1.87 ± 0.46	1.91 ± 0.41	*t* = 1.34	0.181
LDL2 (mmol/L)	3.31 ± 0.89	3.25 ± 0.97	3.32 ± 0.88	t = 1.17	0.243
*75 g OGTT between 24* ~ *28 weeks of pregnancy*					
FBG2 (mmol/L)	4.36 ± 0.41	4.75 ± 0.52	4.30 ± 0.35	*t* = − 13.39	< 0.001
1 h-BG (mmol/L)	7.51 ± 1.64	9.69 ± 1.44	7.14 ± 1.36	*t* = − 27.72	< 0.001
2 h-BG (mmol/L)	6.43 ± 1.28	8.14 ± 1.33	6.15 ± 1.02	*t* = − 22.97	< 0.001
FINS (pmol/L)	61.95 (44.93–83.89)	79.03 (57.11–103.90)	59.65 (43.84–80.78)	*Z* = − 7.85	< 0.001
1 h-INS (pmol/L)	502.34 (347.40–705.24)	610.92 (432.22–887.25)	490.51 (333.17–675.40)	*Z* = − 6.88	< 0.001
1 h-INS (pmol/L)	440.33 (296.94–641.81)	667.60 (505.21–932.13)	415.88 (282.36–579.39)	*Z* = − 12.55	< 0.001
HOMA–IR	1.70 (1.21–2.40)	2.32 (1.64–3.37)	1.63 (1.17–2.26)	*Z* = − 9.61	< 0.001
*Maternal outcomes*					
ICP	31 (1.71)	6 (2.32)	25 (1.60)	*χ*^2^ = 0.32	0.574
Gestational hypertension and pre-eclampsia	165 (9.08)	32 (12.36)	133 (8.53)	*χ*^2^ = 3.94	0.047
Primary cesarean section	639 (35.15)	108 (41.70)	531 (34.06)	*χ*^2^ = 5.69	0.017
Postpartum hemorrhage	83 (4.57)	11 (4.25)	72 (4.62)	*χ*^2^ = 0.07	0.791
Shoulder dystocia or forceps or intermediate caesarean section	67(3.69)	7(2.70)	60(3.85)	*χ*^2^ = 0.82	0.365
*Neonatal outcomes*					
NICU admission	355 (19.53)	54 (20.85)	301 (19.31)	*χ*^2^ = 0.34	0.562
Neonatal jaundice (phototherapy)	292 (16.06)	42 (16.22)	250 (16.04)	*χ*^2^ = 0.01	0.942
Respiratory distress syndrome	22 (1.21)	2 (0.77)	20 (1.28)	*χ*^2^ = 0.15	0.697
Pneumonia	176 (9.68)	29 (11.20)	147 (9.43)	*χ*^2^ = 0.79	0.373
Wet lung	9 (0.50)	4 (1.54)	5 (0.32)	*χ*^2^ = 4.50	0.034
Sepsis	19 (1.05)	1 (0.39)	18 (1.15)	*χ*^2^ = 0.63	0.426
Other infections	37 (2.04)	5 (1.93)	32 (2.05)	*χ*^2^ = 0.02	0.897
Birth weight (g)	3262.5 (2990, 3520)	3250 (2995, 3530)	3270 (2985, 3520)	*Z* = − 0.26	0.792
Macrosomia(BW ≥ 4000 g)	49 (2.70)	9 (3.47)	40 (2.57)	*χ*^2^ = 0.70	0.403
LGA	233 (12.82)	45 (17.37)	188 (12.06)	*χ*^2^ = 5.62	0.018
SGA	130 (7.15)	16 (6.18)	114 (7.31)	*χ*^2^ = 0.43	0.512
AGA	1455 (80.03)	198 (76.45)	1257 (80.63)	*χ*^2^ = 2.43	0.119
Low birth weight at term	26 (1.43)	3 (1.16)	23 (1.48)	*χ*^2^ = 0.01	0.908
Hypoglycemia (< 2.6 mmol/L)	12 (0.66)	1 (0.39)	11 (0.71)	*χ*^2^ = 0.03	0.862
Hypocalcemia	7 (0.39)	2 (0.77)	5 (0.32)	Fisher’s exact	0.262
Gestational age at delivery (weeks)	39.14 (38.43, 39.86)	38.86 (38.14, 39.57)	39.14 (38.43, 39.86)	*Z* = − 4.22	< 0.001
Preterm birth	115 (6.33)	19 (7.34)	96 (6.16)	*χ*^2^ = 0.52	0.471
Placental abruption	23 (1.27)	9 (3.47)	14 (0.90)	*χ*^2^ = 9.83	0.002
Male *n* (%)	958 (52.70)	134 (51.74)	824 (52.85)	*χ*^2^ = 0.11	0.739
Apgar score ≤ 7 at 1 min or 5 min	48 (2.64)	4 (1.54)	44 (2.82)	*χ*^2^ = 1.41	0.235

Bold value means *P* value < 0.05, which indicates statistically significant

*FINS* Fasting insulin, ICP Intrahepatic cholestasis of pregnancy, *LGA* large for gestational age, *SGA* small for gestational age, *AGA* appropriate for gestational age (defined as birth weight between the 10th and 90th percentiles for gestational age), *NICU* neonatal intensive care unit

In addition, the GDM group showed significantly higher rates of adverse perinatal outcomes, such as large-for-gestational-age infants (LGA: 17.4% vs. 12.1%, *p* = 0.018), placental abruption (3.5% vs. 0.9%, *p* = 0.002), gestational hypertension/pre-eclampsia (12.4% vs. 8.5%, *p* = 0.047), primary cesarean delivery (41.7% vs. 34.1%, *p* = 0.017), and neonatal wet lung syndrome (1.5% vs. 0.3%, *p* = 0.034). Notably, while the overall preterm birth rate (< 37 weeks) in the cohort was 6.33%, consistent with national estimates (6.1%, 95% CI 5.1–7.4) [30], no significant difference was observed between GDM and NGT groups (*p* = 0.471). These findings systematically highlight the detrimental effects of GDM on mid-to-late pregnancy metabolic homeostasis and its strong association with adverse maternal–neonatal outcomes, underscoring the imperative for early clinical intervention.

### Key predictors and model performance

Comparative analyses of the three models revealed comparable discriminative performance between Model_2 and Model_3 via DeLong’*s* test (*p* > 0.05, Table 4). However, Hosmer–Lemeshow tests indicated superior calibration for Model_1 (*χ*^2^ = 8.933, *P* = 0.345) and Model_2 (*χ*^2^ = 8.160, *p* = 0.418), while Model_3 showed significant misfit (*χ*^2^ = 20.351, *p* = 0.009). Integrating these results, Model_2 based on LASSO regression with 1SE rule was selected for its balance of parsimony and accuracy.

**Table 4:** Predictive models constructed from three predictor screening strategies were subjected to DeLong’*s* test

Comparison group	Train(AUC, AUC, *p*)	Validation(AUC, AUC, *p*)	All data(AUC, AUC, *P*)
Model_1 vs. Model_2	0.702 vs. 0.721, 0.021*	0.698 vs. 0.705, 0.571	0.702 vs. 0.717, 0.007**
Model_1 vs. Model_3	0.702 vs. 0.727, 0.012*	0.698 vs. 0.670, 0.012*	0.702 vs. 0.719, 0.005**
Model_2 vs. Model_3	0.721 vs. 0.727, 0.302	0.705 vs. 0.670, 0.049*	0.717 vs. 0.719, 0.470

Model_1: MDM, PreBMI ≥ 24, TG/HDL1 ratio ≥ 2.5, Hb1, HbA1c1, FBG1, hGDM

Model_2: MDM, PreBMI ≥ 24, TG/HDL1 ratio ≥ 2.5, Hb1, HbA1c1, FBG1, hGDM, LDL1, Age

Model_3: MDM, PreBMI ≥ 24, TG/HDL1 ratio ≥ 2.5, Hb1, HbA1c1, FBG1, hGDM, LDL1, Age, Parity

^*^p < 0.05, **p < 0.01, ***p < 0.001

Based on the DeLong’*s* test in Table 4 and the multicollinearity test in Table 5, this study empirically validates the key advantages of machine learning methods—particularly LASSO regression—in handling multidimensional clinical data with potentially complex interdependencies. Through its built-in regularization mechanism, LASSO enables automatic and objective variable selection, mitigating overfitting while enhancing model stability and generalization capability. This data-driven automated process proves more efficient than traditional stepwise regression relying on statistical significance thresholds, reliably identifying feature sets with the highest predictive value. Precisely, because of these advantages—especially the optimal balance between parsimony and performance demonstrated under the 1SE criterion—we ultimately selected the LASSO–1SE (*λ* = 0.0239) criterion for predictor selection and constructed an early GDM prediction model. This choice laid a solid foundation for the model’s robust performance and subsequent clinical interpretation.

**Table 5:** GDM prediction model through LASSO regression with the 1SE rule and VIF test results

Predictors	B	SE	Wald	*p*	OR	95% CI	VIF
Age (years)	0.048	0.018	7.441	0.006	1.049	1.014–1.086	1.036
PreBMI ≥ 24 (kg/m^2^)	0.550	0.158	12.087	0.001	1.733	1.271–2.363	1.057
hGDM	1.164	0.412	7.981	0.005	3.203	1.428–7.183	1.022
MDM	0.814	0.273	8.914	0.003	2.257	1.323–3.852	1.013
TG/HDL1 ratio ≥ 2.5	1.298	0.452	8.261	0.004	3.663	1.511–8.879	1.009
Hb1 (g/L)	0.021	0.008	7.154	0.007	1.021	1.006–1.036	1.039
FBG1 (mmol/L)	1.162	0.19	37.247	0	3.198	2.201–4.645	1.059
HbA1c1 (%)	1.037	0.271	14.663	0	2.820	1.659–4.793	1.071
LDL1(mmol/L)	0.207	0.101	4.156	0.041	1.230	1.008–1.500	1.032
constant	− 17.325	1.926	80.89	0	0		

The GDM prediction model was based on Model_2 (LASSO–1SE rule, *λ* = 0.0239) identified nine independent predictors: maternal age, PreBMI ≥ 24 kg/m^2^, hGDM, MDM, TG/HDL ratio, Hb1, FBG1, HbA1c1 and LDL1. These variables exhibited minimal multicollinearity (VIF < 1.1) and significant associations (*p* < 0.05). Key metabolic markers, including FBG1 (OR = 3.20, 95% CI 2.20–4.65) and HbA1c1 (OR = 2.82, 95% CI 1.66–4.79), were the most influential predictors, followed by lipid abnormalities (TG/HDL ratio ≥ 2.5, OR = 3.66) and obesity (PreBMI ≥ 24 kg/m^2^, OR = 1.73). The model exhibited robust discriminative performance in both training (AUC = 0.721, 95% CI 0.679–0.762) and validation cohorts (AUC = 0.705, 95% CI 0.632–0.778), alongside excellent calibration (Hosmer–Lemeshow *p* = 0.510 and 0.385, respectively).This model emphasizes dysglycemia, dyslipidemia, and metabolic history as critical targets for early GDM risk stratification (Table 5).

### Comparative analysis

The GDM prediction model showed good predictive performance with an AUC of 0.717 (95% CI 0.681–0.753) and a high positive predictive value (PPV = 0.923), suggesting strong discrimination and reliability in identifying true positives. It balanced sensitivity (69.7%) and specificity (64.9%), outperforming individual predictors such as HbA1c, FBG and LDL in overall accuracy. By integrating different biomarkers and anthropometric factors, the model enables comprehensive risk stratification, highlighting its clinical utility for targeted early intervention (Table 6).

**Table 6:** Performance metrics (AUC, accuracy, sensitivity, specificity, PPV, NPV, cutoff) of individual predictors and the integrated clinical prediction model

Predictors	AUC (95% CI)	Accuracy	Sensitivity	Specificity	PPV	NPV	Cut off
Age (years)	0.583 (0.546–0.620)	0.710	0.769	0.355	0.878	0.204	33.5
PreBMI ≥ 24 (kg/m^2^)	0.586 (0.555–0.617)	0.751	0.817	0.355	0.884	0.244	
hGDM	0.520 (0.506–0.534)	0.856	0.990	0.050	0.862	0.448	
MDM	0.530 (0.512–0.549)	0.840	0.964	0.097	0.865	0.309	
TG/HDL1 ratio ≥ 2.5	0.515 (0.503–0.527)	0.855	0.991	0.039	0.861	0.417	
Hb1 (g/L)	0.573 (0.536–0.611)	0.512	0.491	0.641	0.892	0.173	126.79
FBG1 (mmol/L)	0.648 (0.610–0.686)	0.667	0.686	0.548	0.901	0.225	4.52
HbA1c1 (%)	0.619 (0.581–0.657)	0.675	0.709	0.471	0.890	0.212	5.39
LDL1(mmol/L)	0.562 (0.523–0.600)	0.623	0.650	0.456	0.878	0.178	2.64
Training	0.721 (0.679–0.762)	0.716	0.735	0.609	0.914	0.290	0.159
Validation	0.705 (0.632–0.778)	0.623	0.606	0.746	0.944	0.210	0.123
The GDM prediction model	0.717 (0.681–0.753)	0.690	0.697	0.649	0.923	0.263	0.14

### Model discrimination and calibration

**Fig. 2:**
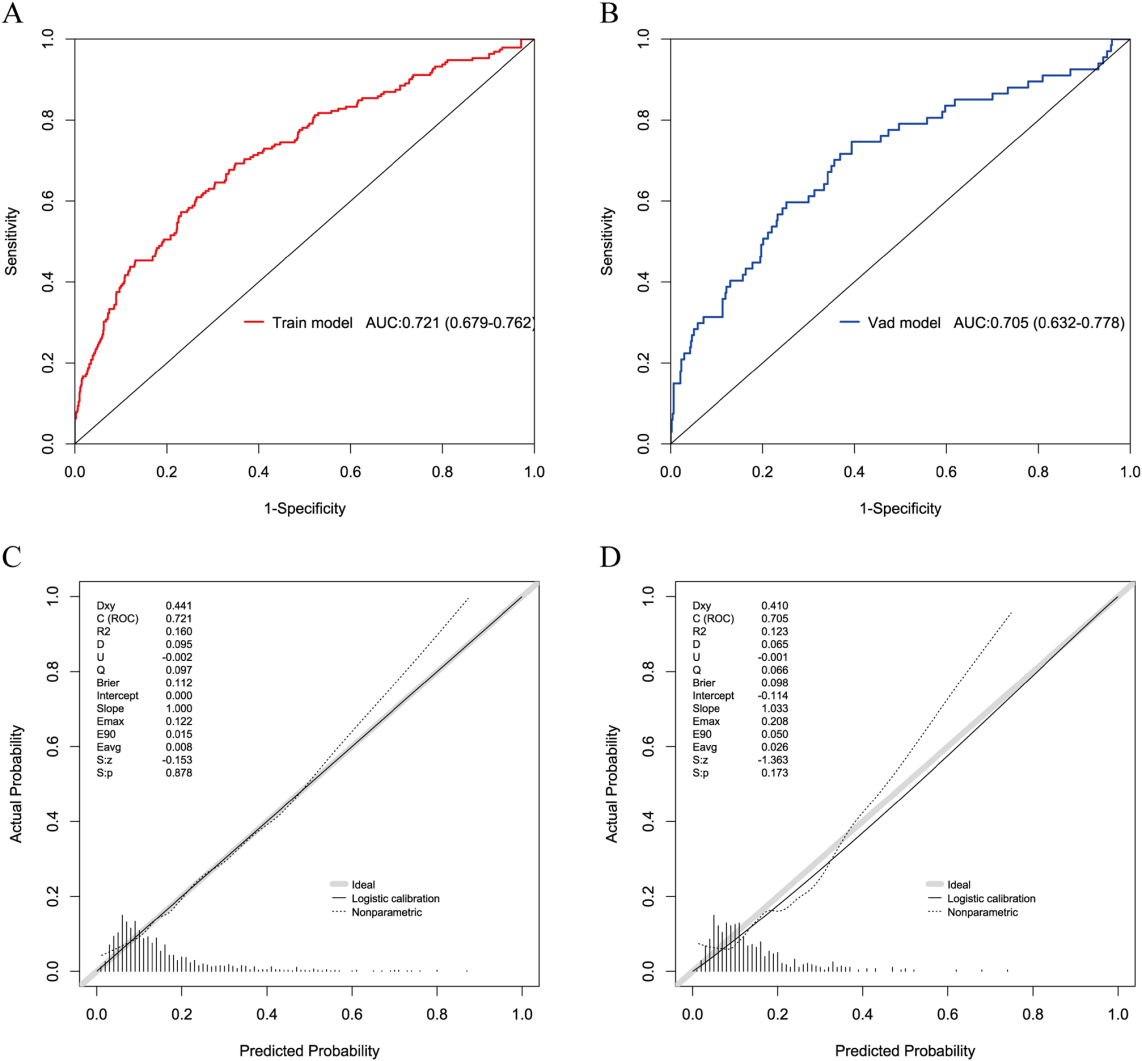
**A**–**B**: ROC curves for the GDM early prediction model with training set AUC = 0.721, 95% CI 0.679–0.762 and validation set AUC = 0.705, 95% CI 0.632–0.778, respectively. **C**–**D**: The calibration curves show the probabilistic accuracy of the GDM prediction model for the training and validation sets, respectively. *y* axis is the actual number of GDM cases, *x* axis is the predicted risk probability, and the solid line reflects the agreement of the model predections of the actual observations

The GDM prediction model exhibited good discrimination, with an AUC of 0.721 in the training set and 0.705 in the validation set (Fig. 2A, B). Calibration performance was excellent, as evidenced by low Brier scores (training: 0.112; validation: 0.098) and near-ideal calibration slopes (training: 1.000; validation: 1.033). Minimal deviations from perfect calibration were observed (Emax: training = 0.122, validation = 0.208), confirming the model’s reliability for clinical risk stratification. Calibration plots demonstrated strong agreement between predicted and observed probabilities in both data sets (Fig. 2C, D).

### Clinical validity and utility

**Fig.3:**
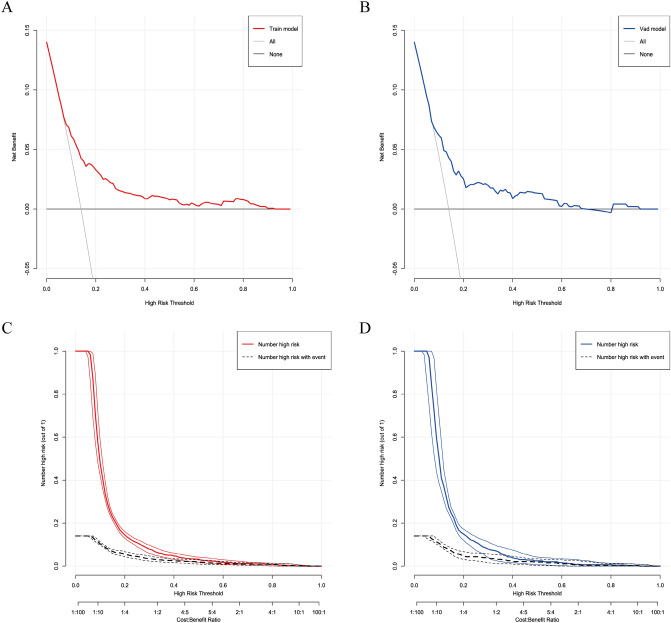
**A**–**B** are the DCA curves for the GDM risk model of the training and validation sets, respectively. The *y* axis estimates the net benefit, the transverse solid line represents the probability of risk that pregnant women have no GDM, and the oblique solid line represents the probability of risk that pregnant women have GDM. **C**–**D** Solid line: Predicted high-risk; dashed line: Actual cases. The term “Number high risk with event” (true positives) reflects the proportion of individuals correctly identified as high-risk who actually develop GDM. At a cost benefit ratio threshold of 1:2–4:5, the model captures 60–80% of high-risk individuals (“Number high risk” − 0.5–0.8) while ensuring that 20–40% of these flagged cases truly develop GDM (“Number high risk with event” − 0.2–0.4). Lower thresholds (eg., 1:100) increase sensitivity (capturing 100% of high-risk cases) but yield poor precision (only 20% true positives), risking unnecessary interventions. Conversely, stricter thresholds (eg., 10:1) prioritize specificity (100% true positives) but miss 60–80% of cases, delaying critical interventions

DCA revealed that the model provided superior net benefit compared to “all” or “none” intervention strategies across risk thresholds of 0.1–0.9 in training set and 0.1–0.7 in validation set, indicating that further diagnosis is beneficial when the predicted risk value is within the above range, and the model has better generalization and consistent performance (Fig. 3A, B).The CIC further validates the clinical utility of the model. For early GDM screening, a threshold close to 1:2–4:5 was used to balance sensitivity (60–80% of cases detected) and precision (20–40% true positives) to optimize timely diagnosis of actionable high-risk pregnancies while avoiding excessive false positives. The stability of the model during the training and validation phases demonstrates its reliability in different clinical settings. This range optimizes timely diagnosis for actionable high-risk pregnancies while avoiding excessive false alarms (Fig. 3C, D).

### Key predictors by LASSO regression

**Fig. 4:**
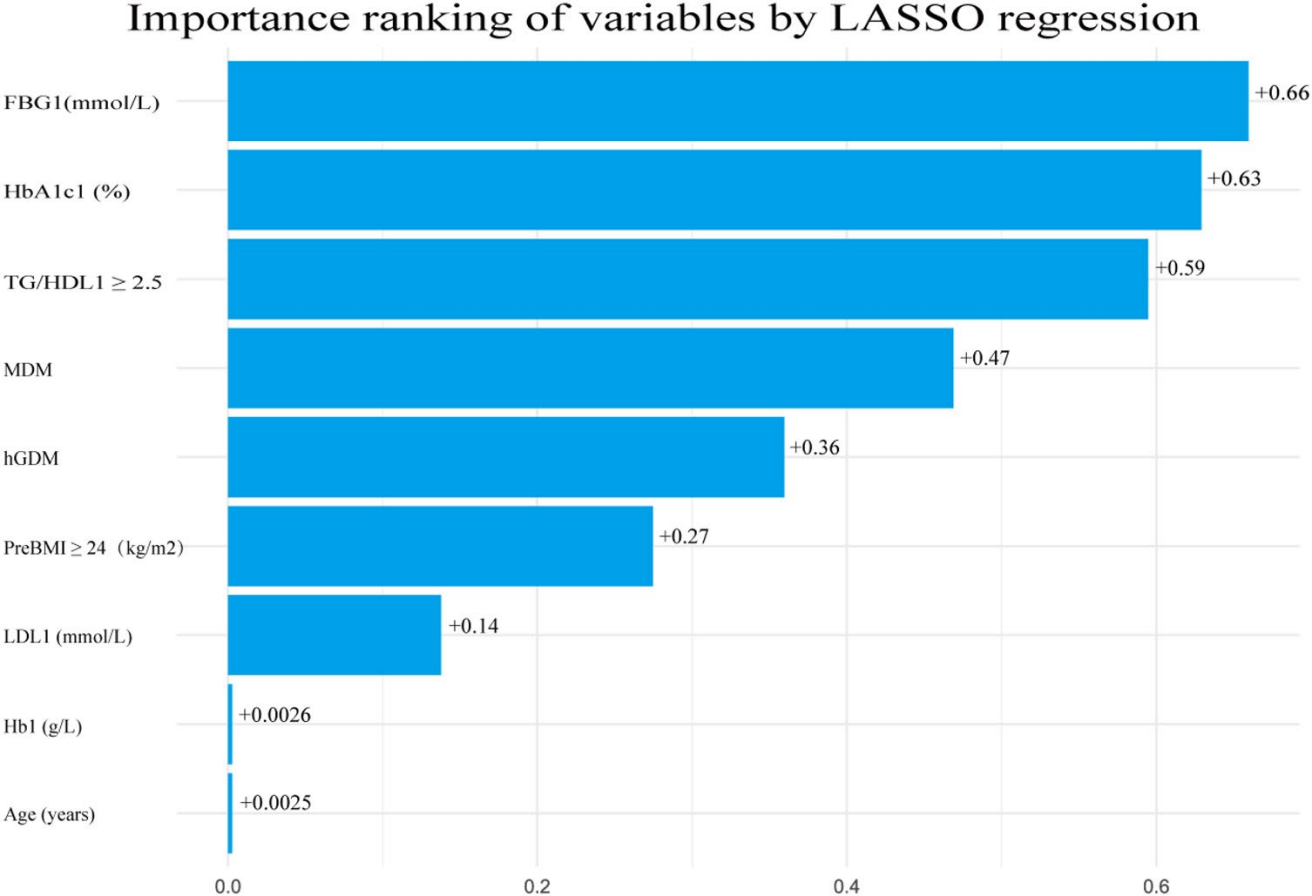
Absolute value of the coefficient

LASSO regression identified FBG1 and HbA1c1 as the most critical predictors of GDM risk, followed by TG/HDL1 ≥ 2.5 and MDM. History of GDM,PreBMI ≥ 24 and LDL1 also had a significant effect, whereas Hb1 and Age were not strongly associated (Fig. 4). These findings highlight the dominant role of glycometabolic and lipid dysfunction in GDM pathogenesis.

### Key predictors and nomogram development

**Fig. 5:**
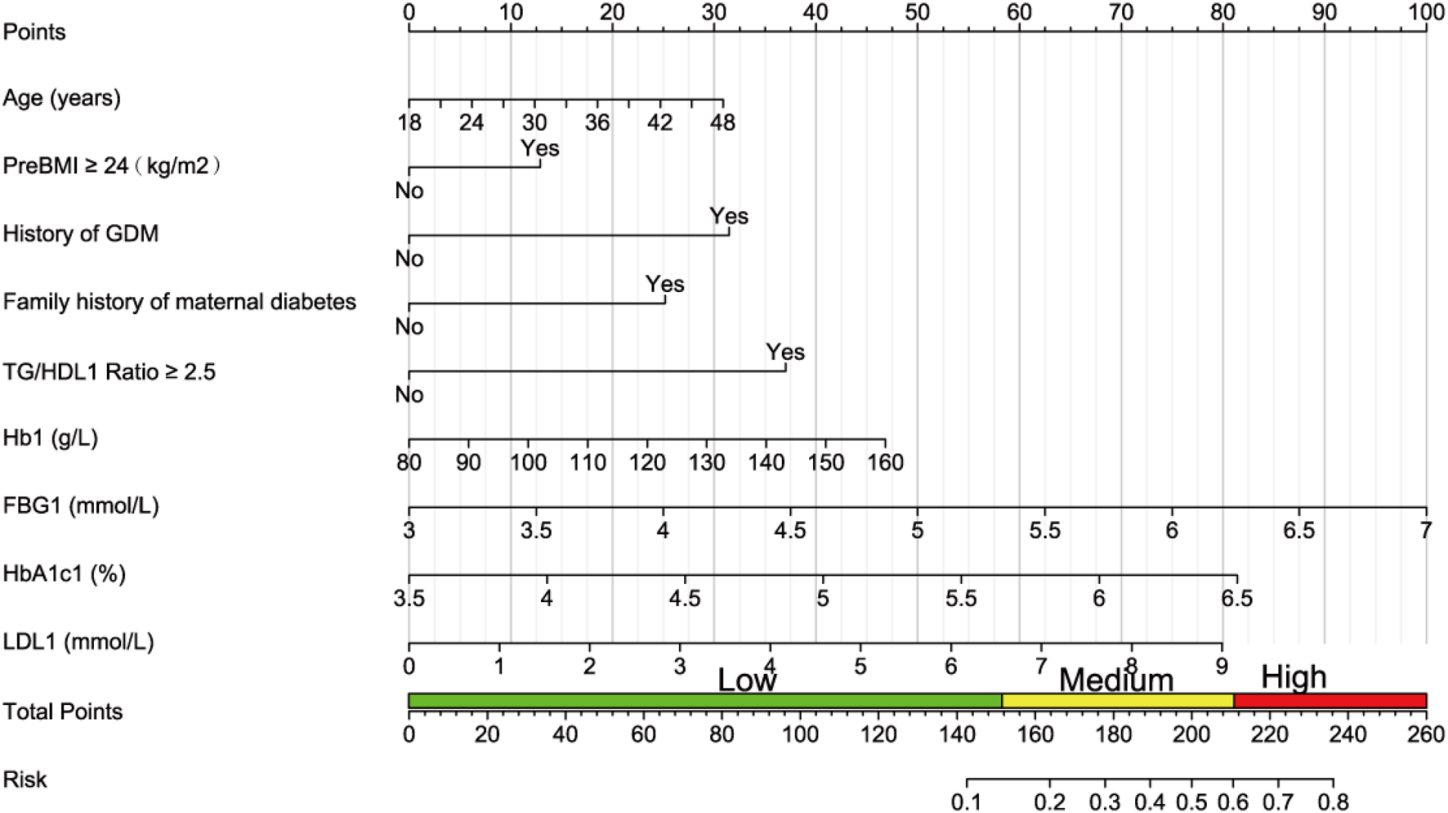
Nomogram to estimate the probability of GDM

The nomogram was developed to translate cumulative scores (0–260) into probabilistic risk estimates (Fig. 5), with glycaemic indices (FBG1, HbA1c1) contributing most significantly. Pre-pregnancy BMI ≥ 24 kg/m^2^, TG/HDL-C ratio ≥ 2.5, and maternal diabetes family history further underscore metabolic and genetic influences. The tool integrates continuous (e.g., Hb1: 80–160 g/L) and binary variables (e.g., hGDM), enabling flexible application across diverse clinical profiles.

### Risk stratification and threshold selection

Early screening for GDM aims to maximize sensitivity and minimize underdiagnosis, so we chose 0.14 as the low-risk threshold based on the cutoff value of the ROC curve, and 0.6 as the medium-to-high-risk threshold in conjunction with the cost–benefit ratio in the CIC charts and the net benefit curve of the DCA, a range that corresponds to a controllable cost of intervention and a high net benefit. The conclusive risk stratification parameters and evidence-based clinical guidelines have been formally established, with comprehensive details provided in Table 7. This stratification balances statistical performance, cost-effectiveness, and clinical practicality, offering a framework for personalized GDM management. Prospective studies are recommended to validate its impact on maternal and neonatal outcomes. In resource-limited settings, the high-risk threshold may be adjusted upward (e.g., > 0.7) to balance sensitivity and cost-effectiveness.

**Table 7:** Final risk stratification thresholds and clinical recommendations

Risk category	Predicted probability	Clinical intervention	Primary objective
Low risk	< 0.14	Routine maternity check-ups, educating patients on self-monitoring of symptoms (e.g., excessive drinking, excessive urination);no additional monitoring	Minimize unnecessary interventions
Medium risk	0.14–0.60	Ensure monthly patient monitoring, simplify screening processes (e.g., fingertip glucose + HbA1c), and provide diet and exercise guidance	Balance early detection and resource allocation
High risk	≥ 0.60	Prioritize OGTT, continuous glucose monitoring (CGM) monitoring, dietician consultations to ensure rapid intervention	Prioritize high-risk cases to prevent adverse outcomes

### Online risk calculator implementation

**Fig. 6:**
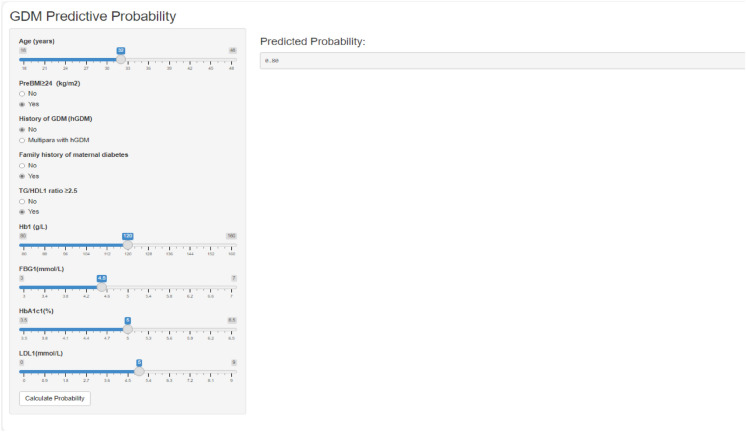
Interface of the online GDM risk calculator (“GDM predictive probability”)

The web-based GDM risk calculator (https://wangxiao0922.shinyapps.io/20250309/) utilizes nine clinical indicators to enable early identification of high-risk pregnancies. It provides real-time risk stratification and specific intervention guidance, such as prompting OGTT for high-risk cases. By shifting initial GDM screening from 24 to 14 weeks of gestation, the tool facilitates lifestyle interventions at least 10 weeks earlier. Its compatibility with electronic health records and user-friendly design support efficient integration into routine prenatal care, significantly optimizing clinical workflow while maintaining data security. Interface of the online GDM risk calculator is depicted in Fig. 6.

## Discussion

GDM arises from a complex interplay of insulin resistance, *β*-cell dysfunction, placental hormone-mediated glucose metabolism disorders, and gut microbiota alterations [2, 19, 31], with placental immune microenvironment also implicated [32]. Emerging evidence highlights roles for inflammatory markers (e.g., CRP and IL-6), adipokines (lipocalin, leptin) [19, 33], metabolomic profiles (plasma branched-chain amino acids, free fatty acids) [34], proteomic markers (IGFBP0) [35, 36], and genetic polymorphisms [21], while imaging techniques such as subcutaneous/visceral fat ultrasound assessment (SAT/VAT) [37] and continuous glucose monitoring [38, 39] enhance risk stratification in high-risk populations. Current GDM prediction models face an “accuracy-practicality” trade-off: simplified models based on basic indicators (e.g., age and PreBMI) exhibit limited discrimination (AUC 0.69) [40], whereas advanced models integrating metabolic markers (e.g. OGTT), genomics, or machine learning achieve higher AUC (0.82–0.87) [21, 41] but suffer from high costs and poor clinical feasibility.

Our LASSO-derived nomogram addresses these limitations by incorporating nine accessible predictors: age ≥ 33.5 years (linked to age-related insulin resistance) [2, 42], PreBMI (chronic inflammation and obesity-driven insulin resistance) [23, 42, 43], prior GDM (OR 10.4 for recurrence; tenfold elevated postpartum T2DM risk) [2, 11, 44], maternal diabetes history (the ORs for this study were 2.257; mitochondrial DNA mutations and epigenetic programming) [45–48], early HbA1c (threshold 5.2–5.6%, reflecting pregnancy-specific glycemic dynamics, possibly related to haemodilution and changes in iron metabolism during pregnancy) [39] [49, 50], FBG(early pregnancy FBG is an independent risk factor for GDM) [40, 51, 52], TG/HDL ratio ≥ 2.5 (lipid-driven insulin resistance and systemic inflammatory response) [25, 53], LDL (synergistic lipid-inflammatory effects) [54, 55], and Hb (threshold effect via oxidative stress and iron metabolism) [56–58]. The model demonstrated moderate discrimination (AUC 0.721 training, 0.705 validation) with robust calibration (Hosmer–Lemeshow *p* = 0.418) and clinical utility across risk thresholds (DCA and CIC-confirmed). Key strengths include cost-effectiveness, interpretability, and adaptability to resource-limited settings via a web-based nomogram.

Notably, lipid indices (TG/HDL, LDL) outperformed traditional obesity-centric metrics, particularly in Asian populations with lower baseline adiposity [25, 53]. Maternal diabetes history exhibited stronger predictive power than paternal history (*p* > 0.7 for FDM), underscoring intrauterine epigenetic programming as a critical pathway [45–48]. While HbA1c and FBG provided synergistic glycemic insights, their pregnancy-specific thresholds require validation against erythrocyte turnover dynamics and hemodilution effects [39, 50].The web tool’s integration with routine prenatal data enables real-time risk stratification without additional costs, addressing a critical gap in low-resource settings.

This study has several limitations. First, due to its retrospective design and reliance on routine electronic medical records, we were unable to directly obtain and quantify key lifestyle variables during pregnancy, such as detailed dietary patterns, physical activity frequency, and intensity. To partially compensate for this, we used region (as a rough proxy for dietary differences between northern and southern China) and educational attainment (as a general indicator of health awareness and behavior) for approximate assessment. However, it must be acknowledged that these proxy variables cannot accurately reflect individual energy intake and expenditure, potentially introducing residual confounding. Second, although we calculated the rate of weight gain in early pregnancy (VT1) to reflect initial gestational weight change, this variable did not show a statistically significant difference between the GDM and non-GDM groups in our cohort, suggesting its limited independent predictive value and indicating a potential need for more refined anthropometric measures (e.g., waist-to-hip ratio). These data-level constraints may partially explain why the model's predictive performance (AUC = 0.717) did not achieve higher levels.

A critical and inherent limitation is the model's uncertain generalizability. Developed from a single-center Shanghai cohort, its core predictive strength lies in the biological universality of established risk factors (e.g., pre-pregnancy BMI, age, family history of diabetes, FBG, and lipid profiles). While this provides a rationale for testing in other settings, its performance may be limited in populations with significantly different genetic backgrounds, lifestyle patterns (e.g., diet and physical activity), or healthcare practices, which could alter the specific weight and calibration of model variables. Therefore, the model should be viewed as a promising but require validation framework rather than a directly deployable tool for dissimilar populations.

To address these limitations and advance the model toward responsible clinical application, we propose a focused future research agenda structured around three sequential goals:

- Prospective data enrichment and model refinement: Future studies should adopt a prospective design to systematically collect detailed lifestyle data using validated tools, such as food frequency questionnaires, physical activity scales, or wearable devices. Concurrently, the integration of accessible, low-cost biomarkers (e.g., serum adiponectin and C-reactive protein) and the exploration of dynamic clinical indicators—such as the early pregnancy systolic blood pressure growth rate, suggested by our findings—should be investigated to enhance the model's discriminatory ability and biological plausibility.
- Rigorous multicenter external validation and calibration: A pivotal next step is to evaluate the model's transportability through prospective, multicenter validation. We recommend an initial collaborative study across 3–5 medical centers in China that represent major geographic and socioeconomic regions (e.g., Northeast, North, South, Northwest, and Southwest). This study must employ standardized protocols for data collection and GDM diagnosis (preferably IADPSG criteria). The analysis will not only test the model's overall performance but also conduct subgroup analyses (e.g., by region and ethnicity) to identify where and for whom the model works, leading to necessary recalibration or adjustment for specific subpopulations.
- Clinical implementation science evaluation: Following successful validation, research should shift toward implementation. This involves packaging the validated model into user-friendly clinical tools (e.g., web calculators and EHR integrations) and conducting pilot studies to evaluate its real-world feasibility, clinician acceptance, impact on high-risk patient identification, and ultimately, its effect on promoting timely intervention within routine prenatal care workflows.

By following this logical progression—from enriching the model’s inputs, to rigorously testing its generalizability, and finally to assessing its practical utility—future work can systematically transform this proof-of-concept framework into an evidence-based tool suitable for broader application.

## Conclusion

This GDM prediction model integrates maternal metabolic indicators and obstetric history into a cost-effective stratification tool that enables automated risk scoring and clinical decision support through integration with electronic medical record systems. An online calculator provides real-time, user-friendly risk stratification and seamless integration with antenatal management to provide actionable guidance for timely intervention. To maintain clinical utility, the model requires regular calibration to optimize risk thresholds. Future studies should validate its generalizability, integrate emerging biomarkers such as immune-inflammatory markers to enhance predictive efficacy, and evaluate scalable early intervention strategies in perinatal care. By integrating predictive analytics with clinical workflow, this approach advances precision prevention while ensuring accessibility across different healthcare settings.

## Data Availability

Data cannot be shared publicly due to patient privacy protections. Findings are fully supported by data within the manuscript. Restricted data may be requested from the corresponding author (xuxm11@163.com) with a signed data-sharing agreement.The R code used for LASSO regression, model development, validation, and generation of nomograms and performance plots is publicly available via the GitHub repository at: [https://github.com/xiaowang-0922/GDM/commit/24bb7d8574d953b28ee6141c5ab0d32a96124d90].
